# Sleep Disorders Associated With Alzheimer's Disease: A Perspective

**DOI:** 10.3389/fnins.2018.00330

**Published:** 2018-05-31

**Authors:** Anna Brzecka, Jerzy Leszek, Ghulam Md Ashraf, Maria Ejma, Marco F. Ávila-Rodriguez, Nagendra S. Yarla, Vadim V. Tarasov, Vladimir N. Chubarev, Anna N. Samsonova, George E. Barreto, Gjumrakch Aliev

**Affiliations:** ^1^Department of Pulmonology and Lung Cancer, Wroclaw Medical University, Wroclaw, Poland; ^2^Department of Psychiatry, Wroclaw Medical University, Wroclaw, Poland; ^3^King Fahd Medical Research Center, King Abdulaziz University, Jeddah, Saudi Arabia; ^4^Department of Neurology, Wroclaw Medical University, Wroclaw, Poland; ^5^Facultad de Ciencias de la Salud, Universidad del Tolima, Ibagué, Colombia; ^6^Department of Biochemistry and Bioinformatics, School of Life Sciences, Institute of Science, Gandhi Institute of Technology and Management University, Visakhapatnam, India; ^7^Institute for Pharmaceutical Science and Translational Medicine, Sechenov First Moscow State Medical University, Moscow, Russia; ^8^Institute of Physiologically Active Compounds of the Russian Academy of Sciences, Chernogolovka, Russia; ^9^Departamento de Nutrición y Bioquímica, Facultad de Ciencias, Pontificia Universidad Javeriana, Bogotá, Colombia; ^10^Instituto de Ciencias Biomédicas, Universidad Autónoma de Chile, Santiago, Chile; ^11^GALLY International Biomedical Research and Consulting LLC, San Antonio, TX, United States; ^12^School of Health Science and Healthcare Administration, University of Atlanta, Johns Creek, GA, United States

**Keywords:** AD, diagnosis, sleep disorders, disturbance, sleep-rhythm

## Abstract

Sleep disturbances, as well as sleep-wake rhythm disturbances, are typical symptoms of Alzheimer's disease (AD) that may precede the other clinical signs of this neurodegenerative disease. Here, we describe clinical features of sleep disorders in AD and the relation between sleep disorders and both cognitive impairment and poor prognosis of the disease. There are difficulties of the diagnosis of sleep disorders based on sleep questionnaires, polysomnography or actigraphy in the AD patients. Typical disturbances of the neurophysiological sleep architecture in the course of the AD include deep sleep and paradoxical sleep deprivation. Among sleep disorders occurring in patients with AD, the most frequent disorders are sleep breathing disorders and restless legs syndrome. Sleep disorders may influence circadian fluctuations of the concentrations of amyloid-β in the interstitial brain fluid and in the cerebrovascular fluid related to the glymphatic brain system and production of the amyloid-β. There is accumulating evidence suggesting that disordered sleep contributes to cognitive decline and the development of AD pathology. In this mini-review, we highlight and discuss the association between sleep disorders and AD.

## Introduction

Alzheimer's disease (AD) is the most common cause of dementia, and its etiology is multifactorial (Chibber et al., 2016). The primary event in AD is the accumulation of amyloid-β (Aβ) in the brain (Karran et al., 2011). Abnormal deposition of Aβ triggers a cascade of events leading to neuroinflammation and neuronal cell death (Wyss-Coray and Mucke, 2002; Selkoe and Hardy, 2016). As a consequence, clinical manifestations of AD, mainly impaired cognitive function, develop progressively over 15 years since the beginning of accumulation of Aβ (Sen et al., 2017). Neuropathological modifications may develop progressively for several decades and during this preclinical period of AD, mild cognitive impairment (MCI) may occur (Drago et al., 2011; Bhat et al., 2017). In over 90% of cases, AD begins after the age of 65 as a sporadic form of dementia (Prince et al., 2013; Ashraf et al., 2016). Of late, AD has been found to coexist with other chronic diseases like cancer, diabetes, and cardiovascular diseases (Aliev et al., 2014; Jabir et al., 2015; Rizvi et al., 2015; Ashraf et al., 2016). This has opened up new dimensions of AD diagnosis and therapy based on proteomics (Ashraf et al., 2014, 2015) and nanotechnology (Soursou et al., 2015; Ansari et al., 2017).

**Figure 1:**
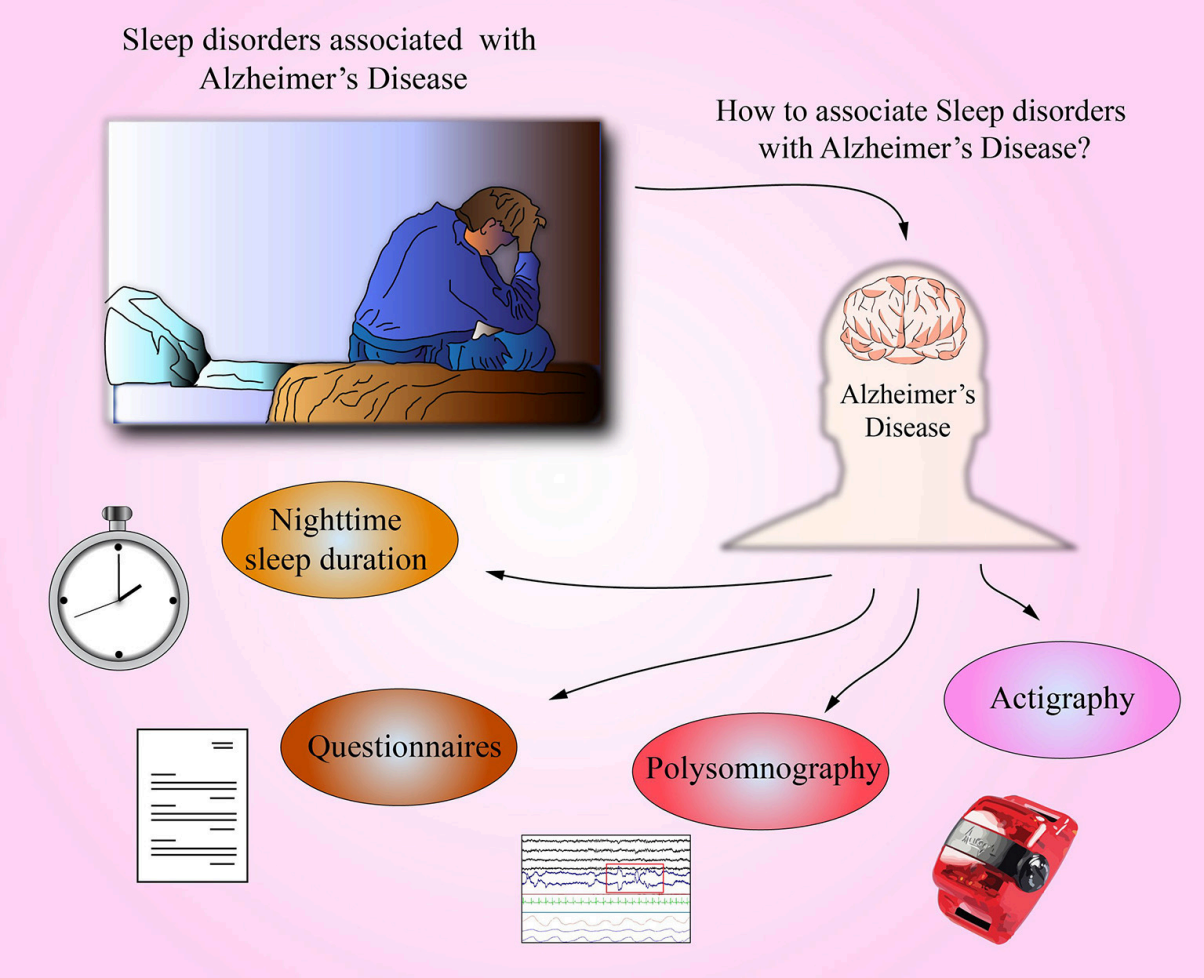
Identification of sleep disorders and possible relationship with AD. Several sleep conditions may indicate the risk of undergo AD; for example, nighttime sleep (<5 h) is linked in many epidemiological studies as a risk factor for AD. Additionally, it is possible to qualitatively survey for sleep quality by means of questionnaires i.e., Athens Insomnia Scale or Pittsburg Sleep Quality Index. Polysomnography is a procedure to diagnose sleep disorders, and in AD patients, it is observed prolonged sleep latency and increased number of arousals. Finally, some portable equipment like Actigraph can provide key data of sleep quality by measuring the wakefulness and sleep activity in several days (10 days registration).

In AD, and likely in other neurodegenerative diseases, sleep disorders appear early (Dos Santos et al., 2014, 2015). Although the time meant for sleeping extend during the day, sleeping and waking rhythms are disturbed (McCurry et al., 1999). Common symptoms include difficulties in falling asleep, arousal at night, repeated awakenings and waking up too early in the morning, and sleepiness and frequent naps during the day (McCurry and Ancoli-Israel, 2003; Most et al., 2012). Sleep disorders can be an important diagnostic indication that foreruns development of AD's pathological disorders in the form of Aβ deposition in the brain and during dementia onset (Lim et al., 2013; Spira et al., 2013). Sleep disorders worsen as the disease progresses (Bliwise et al., 1995), and their considerable intensification in the late stage of the disease is a strong predictive factor for mortality (Spalletta et al., 2015). In the present mini-review, we discuss the association of sleep disorders in AD (Figure 1).

### Night-time sleep duration and AD

Results of clinical and epidemiological studies regarding a connection between night-time sleep duration and risk of AD are not equivocal. Although a prospective, 2-years self-reporting study in 1,844 community-dwelling women in the age ≥70 has shown that women sleeping ≤5 h per night had poorer cognitive abilities than women sleeping longer, though the difference was small (Tworoger et al., 2006). Similar results were found in the Spanish study in 3,212 people at the age of ≥60, where no correlation was found between shortened (<7 h) sleep time (according to self reported data) and cognitive disorders, determined by Mini-Mental State Examination (MMSE) questionnaire (Faubel et al., 2009). However, among persons sleeping longer than 7 h, a statistically significant (*p* < 0.001) trend was proved, indicating that the longer the sleep (more than 7, 8, 9, 10, or 11 h), the worse the cognitive abilities are (Faubel et al., 2009). A progressive study based on the Pittsburgh Sleep Quality Index and encompassing 1,664 persons at the age of 65 years without cognitive disorders during a 1-year observation found that extended sleep time (>9 h) among women and shortened sleep time (<5 h) among men correlated with cognitive impairment (OR 3.70; 95% CI 1.49–9.17 and OR 4.95; 95% CI 1.72–14.27, respectively) (Potvin et al., 2012). Further, in a study in 298 women without dementia at the age of 82.3 ± 3.2 years, a total sleep time (TST) during polysomnography (PSG) did not show correlation with the degree of cognitive impairment (Yaffe et al., 2011).

Based on results obtained in empirical study conducted among healthy men in middle age, it has been hypothesized that Aβ_42_ increases with chronic sleep deprivation (Ooms et al., 2014). In this study, encompassing men, aged 40–60 years, without cognitive impairments, 13 persons have not been allowed to fall asleep at night, while the other 13 persons were permitted to unlimitedly long sleep. In both groups, a concentration of Aβ_42_ was measured in cerebrospinal fluid (CSF) collected in the evening and in the morning. In persons who slept at night, a 6% decrease of Aβ_42_ level in the morning compared to evening hours had been observed (95% CI [0.94, 49.6], *p* = 0.04). However, among those who did not sleep, the physiological decrease of Aβ_42_ level in the morning hours had not been noticed. Thus, observed increased levels of Aβ_42_ in CSF after sleep deprivation might indicate a higher risk of development of AD. Moreover, there was a correlation between total sleep duration and maximal decrease of Aβ_42_ (*r* = -0.5, *p* = 0.04).

### Diagnostics of sleep disorders based on questionnaires in AD patients

In patients with AD, the diagnostics of sleep disorders based on specific questionnaires is difficult due to cognitive impairment affecting reliability of self-report measures of sleep. It may happen that patients who suffer from severe difficulties in falling asleep and frequent awakenings at night do not complain of insomnia at all (Most et al., 2012). The study comparing results of sleep questionnaires, such as Pittsburgh Sleep Quality Index, the Sleep Disorders Questionnaire, and the Athens Insomnia Scale, with the results of actigraphy in 55 patients with AD and 26 controls revealed limited value of those sleep questionnaires in early and moderate AD stage (Most et al., 2012). Based on the results obtained in the questionnaires, it has been found that sleep disorders occurred in 24.5% of patients with mild to moderate form of AD (Moran et al., 2005). However, it appears that sleep disorders occur much more frequently in the course of this disease (Zhao et al., 2016).

### PSG in the diagnostics of sleep disorders in AD patients

PSG is a basic and objective method of diagnosing sleep disorders. In the patients with AD, PSG usually shows prolonged sleep latency, i.e., time taken to fall asleep (McCurry and Ancoli-Israel, 2003). Indeed, increased number of awakenings and lengthened time of wakefulness after sleep onset causes reduced sleep efficiency (Bliwise, 1993; Rauchs et al., 2008).

The number of sleep cycles remains unchanged (Petit et al., 2004), but duration of both rapid eye movement (REM) sleep and deep sleep (N3) is usually shortened (Maestri et al., 2015). However, in AD, recognition of sleep stages—especially stage N3—is frequently difficult, because usually in electroencephalogram (EEG) recordings there are generalized slow waves (0.5–2 Hz) of low amplitude during both sleep and wakefulness (Petit et al., 2004; Peter-Derex et al., 2015). Also, during REM sleep, an increased amount of delta and theta waves and reduced number of faster α and β waves can be observed (Hassainia et al., 1997). Reduced activity of EEG is considered as a biological marker of AD (Prinz et al., 1982). Based on the measurements of the cyclic alternating pattern (CAP) in the PSG recordings, sleep instability was found both in the subjects with MCI (age 68.5 ± 7.0 years) and—to a greater extent—in patients with mild AD (age 72.7 ± 5.9 years) as compared to healthy persons without cognitive impairment (age 69.2 ± 12.6 years) (Maestri et al., 2015). In this study, encompassing 33 subjects in the three equally numerous groups, PSG revealed abnormalities in the microstructure of sleep, as indicated by decreased CAP rate and slow components of CAP. Thus, PSG abnormalities could serve as a potentially useful marker of neurodegeneration in subjects with cognitive impairments. Disordered sleep structure correlates with a degree of cognitive abilities impairment, including those assessed by MMSE. The correlations of cognitive impairments and sleep structure abnormalities in PSG recordings were found in a study of 48 patients with AD (21 patients with mild AD and 27 patients with moderate to severe AD) (Liguori et al., 2014). In this study, abnormalities of macrostructural sleep variables in PSG were more pronounced in patients with poorer cognitive function (MMSE score < 21).

Abnormalities in the PSG recordings were also noted in patients with preclinical AD. In a previous study of 25 subjects with MCI (age 70.5 ± 6.8 years, MMSE score 26.7 ± 2.4), higher density of arousals during slow wave sleep and decreased percentage of REM sleep during total sleep time as compared with healthy subjects in similar age were found (0.09 ± 0.11 vs. 0.19 ± 0.10; *p* < 0.01 and 14.7 ± 3.7 vs. 10.1 ± 5.4; *p* < 0.007, respectively) (Hita-Yañez et al., 2013).

In patients with amnestic MCI—who constitute a group of increased risk of progression to AD—abnormalities in the sleep structure were also observed. In a study of 8 amnestic MCI patients (age 72.1 ± 5.1), as compared with 16 age-matched healthy adults, there were fewer sleep spindles, shortened SWS and lower delta and theta power (Westerberg et al., 2012). However, PSG has limited usage for patients with AD. Its limitations rely in the fact that most patients with AD—especially in advanced stage—are not able to cooperate during the examination and do not tolerate any electrodes and sensors on the skin (Peter-Derex et al., 2015).

### Actigraphy to examine sleep disorders in patients with AD

Actigraphy turned out to be appropriate method to examine sleep disorders in AD (Ancoli-Israel et al., 1997; Most et al., 2012). Prospective study based on actigraphy (10 days registration) conducted in 737 men and women at the age of 81.6 ± 7.2 has shown that after an average 3.3 years, risk of symptoms of AD was 1.5 times higher in subjects with high sleep fragmentation as compared to subjects with slight sleep fragmentation (Lim et al., 2013). Sleep studies on the basis of actigraphy (2 weeks registration) have been conducted in 142 persons without cognitive disorders at the age of ≥45 years (Ju et al., 2013). In this group, more than half of the persons (54.2%) were at the age >65 years, including 18 persons (12.7%) at the age over 75 years. TST (i.e., the amount of sleep) and the percentage of sleep in the time spent in bed (i.e., efficiency of sleep) have been determined. In this study, it has been arbitrarily stated that sleep efficiency <75% showed worse sleep quality, and correlation of quantity and quality of sleep and the level of Aβ_42_ in CSF have been evaluated. No differences in TST have been found in persons with decreased and normal levels of Aβ_42_ in CSF. In 32 persons (22.5%) on the basis of lowered level of Aβ_42_ in CSF (≤ 500 pg/ml), pre-clinical form of AD was diagnosed. In this group, quality of sleep was worse than among other persons (80.4 vs. 83.7%, *p* = 0.04) (Ju et al., 2013). High proportions of the persons studied were at the age > 65 years, indicating possible influence of this factor on the obtained results. Additionally, it should be stated that Aβ_42_ thresholds in CSF are not clearly defined in cognitively healthy persons. For persons in pre-clinical stage of AD, as defined on the basis of decreased Aβ_42_ levels in the CSF, at least 3 naps in a week have been noted, i.e., more than for persons without signs of amyloid deposition (31.2 vs. 14.7%; *p* = 0.03). The results of the study have confirmed that the most important sleep disorder in AD is sleep fragmentation, causing worsening of the sleep quality (Ju et al., 2013). However, there might be bidirectional influence of amyloid deposition on sleep, and the authors indicate both the possibilities that Aβ_42_ interferes with neuronal function related to sleep-wake cycle and that sleep disturbances contribute to amyloid deposition.

### Breathing disorders during sleep in AD patients

Sleep disorders in AD can be caused by breathing disorders during sleep and among them by repetitive obstructive sleep apnea (OSA). However, a correlation between breathing disorders during sleep and AD is not well explained. OSA syndrome occurs—similarly to AD—more often in older patients (Ancoli-Israel et al., 1991). The main risk factor of OSA syndrome is overweight or obesity. Obesity is diagnosed when body mass index (BMI) exceeds 30 kg/m^2^. In the population of obese persons, with BMI >30 kg/m^2^, OSA risk is about 20–40% (Saint Martin et al., 2015). A connection of AD with obesity is complex. High BMI in the middle of life relates with increased risk of AD in later life, while high BMI in later life is associated with lower risk (Whitmer et al., 2005; Emmerzaal et al., 2015). In a prospective study, where correlation between AD and body weight was analyzed, it has been stated that among patients who were obese at 50 years old, risk of AD was higher (HR 1.39; 95% CI 1.03–1.87) in comparison to patients with normal weight. A reversed correlation has been found by analyzing BMI in later life (i.e., in 65 years of age): among obese patients, the risk of AD was lower (HR 0.63, 95%CI 0.44–0.91) when compared to patients with normal BMI (Fitzpatrick et al., 2009). In the other prospective study comprising 1,394 persons, who at the age of 50 did not show any cognitive disorders, were followed-up for 14 years, and results showed that 142 of them had developed AD. It has been stated that among persons who were obese, upon the beginning of trial, AD developed, on the average, 6.7 months earlier (Chuang et al., 2016). Decrease of BMI before AD (about 0.21 kg/m^2^ annually, otherwise about 0.6 kg annually for a person being 1.7 m tall) and stabilization or increase in BMI after the appearance of clinical symptoms of the disease were observed (Gu et al., 2014). Unfortunately, in the above cited studies on the link between obesity and AD risk, sleep breathing disorders have not been assessed. However, as there is increasing incidence of sleep apneas and hypopneas with increasing weight and sleep breathing disorders, and these variables should be taken into the consideration. According to some reports, breathing disorders during sleep occur more often in AD than among persons without dementia (Hoch et al., 1986; Gehrman et al., 2003; Janssens et al., 2009; Kinugawa et al., 2014). In other studies, differences in the frequency of sleep breathing disorders in PSG studies in AD, in comparison to control groups, were minor (Bliwise, 2002; Moraes et al., 2008); while in some studies these differences have not been noticed at all (Bliwise et al., 1989). A possibility of participation of breathing disorders during sleep in etiology of AD is considered by inflammation states, oxidative stress and hypoxemia being caused by them (Dyken et al., 2004; Daulatzai, 2012, 2013). Moreover, breathing disorders during sleep may contribute to progress of AD-related vascular changes. For example, in the study of Buratti et al. (2014) the intima-media thickness (IMT) and cerebrovascular reactivity to hypercapnia based on a breath-holding index (BHI) have been compared in groups of patients with and without OSA syndrome in the course of AD (Buratti et al., 2014). It has been stated that incorrect values of examined parameters (IMT > 1.0 mm, BHI < 0.69) occurred more often in patients with OSA syndrome than in patients without breathing disorders during sleep (HR respectively, 2.98; 95% CI: 1.37–6.46, *p* < 0.05 and 5.25; 95% CI: 2.35–11.74, *p* < 0.05). Indirectly, a correlation between sleep fragmentation and appearance of cognitive disorders was found in the observations conducted in 298 women at the age of 82.3 ± 3.2 years, who were diagnosed with OSA syndrome. Repeated breathing disorders in this syndrome caused sleep fragmentation, because arousals finish the periods of apneas and hypopneas. A prospective study revealed that after 5 years, the risk of mild cognitive disorders or dementi clearly grows (OR 2.04; 95% CI 1.10–3.78) together with increase of apneas and hypopneas frequency (Yaffe et al., 2011).

In a previous study encompassing 59 patients with dementia (MMSE 20.1 ± 6.6), who underwent PSG, OSA syndrome (moderate or severe form) was diagnosed in almost half of the patients (49%). It has been stated at the same time that risk of excitation at night was the smallest in the patients with high apnoe/hypopnoe index (AHI), i.e., with more severe OSA syndrome (Rose et al., 2011). It has been also proved that for some patients with mild or moderate form of AD, prevention of obstructive breathing disorders during sleep with continuous positive airway pressure (CPAP) can slow down development of dementia (Cooke et al., 2009). For instance, in the group of 23 patients with mild and moderate form of AD (MMSE > 15), as well as with severe form of OSA syndrome (AHI ≥ 30), cognitive disorders were compared after 3 years among patients who used or did not use CPAP and a reduction in the rate of cognitive disorders decline has been stated—measured in MMSE scale—in the group using CPAP [−0.7 (90% CI −1.7; +0.8 vs. −2.2 (90% CI 3.3–1.9); *p* = 0.013] (Troussière et al., 2014).

**Figure 2:**
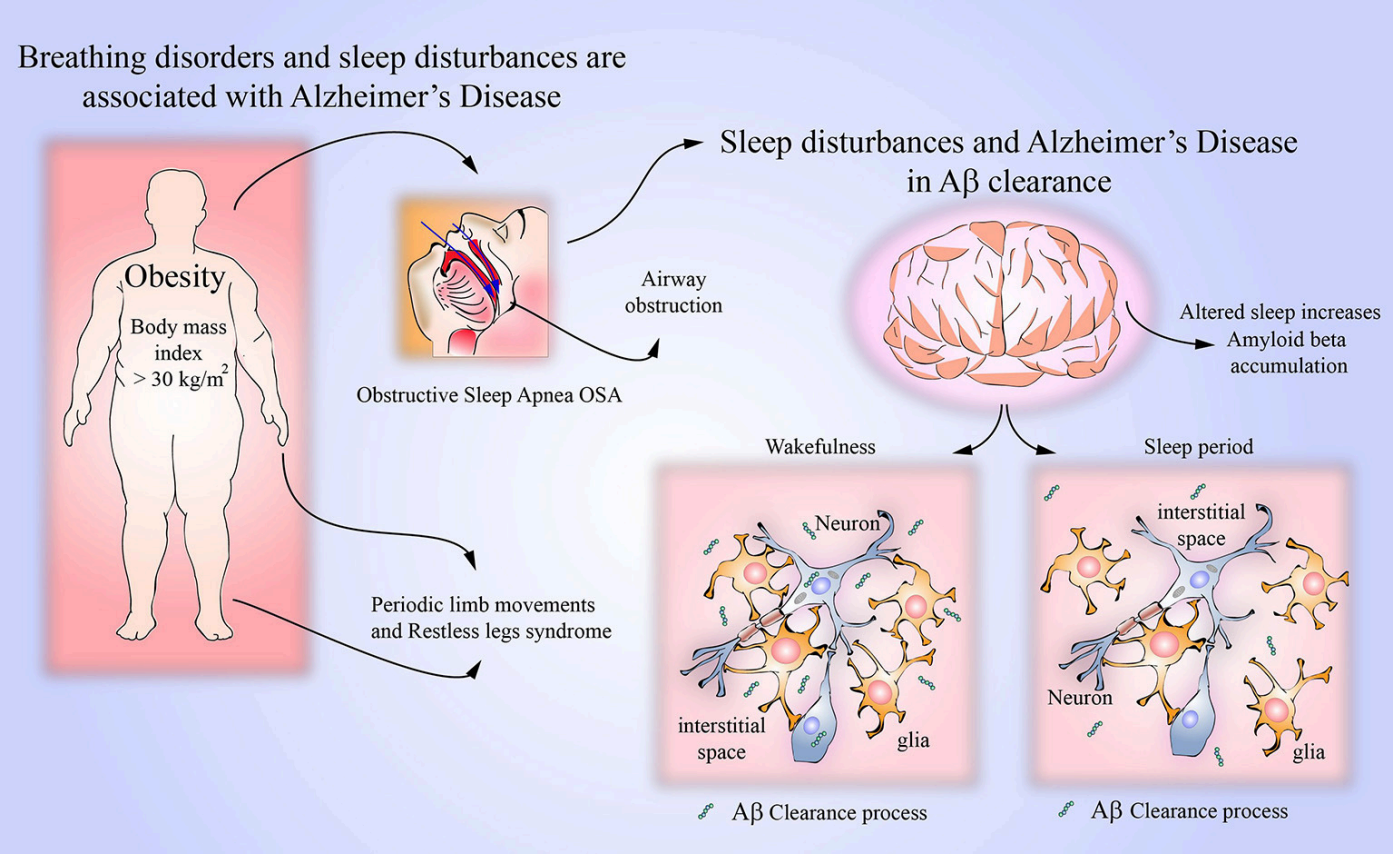
Breathing disorders and amyloid beta clearance. It has been observed that sleep disorders in AD can be caused by obstructive sleep apnea (OSA). OSA is caused by an obstruction of respiratory airways, and its main risk overweight in obesity. Prospective studies have shown that patients with OSA may develop AD by several mechanisms such as neuroinflammation, oxidative stress and hypoxemia. Interestingly, clinical conditions like periodic limb movements and restless legs syndrome, which also lead to sleep disturbances, may be associated to AD. The removal of metabolites and neurotoxic compounds via glymphatic system is a function regulated by sleep and wakefulness activity. It is known that soluble amyloid beta can be cleared from the CNS parenchyma using the glymphatic system. Sleep may influence the clearance of amyloid beta due to the increased brain's interstitial space possibly by the shrinkage of astroglial cells.

There is evidence indicating a link between OSA and AD (Figure 2). In a recently published study, it has been shown that OSA might induce early changes in CSF Aß 42 concentrations (Liguori et al., 2017). In this study, CSF Aß_42_ concentrations were measured in 25 moderate or severe OSA patients with apnea and hypopnea index > 15/h, in 10 OSA patients treated with continuous positive airway pressure (CPAP, method of choice eliminating sleep apneas) and in 15 controls. In untreated OSA patients, CSF Aß_42_ concentrations were lower than in controls and lower than in CPAP treated OSA patients. Additionally, in OSA patients, a correlation between CSF Aß_42_ concentrations and arterial oxygen saturation during sleep was found, thus confirming the influence of the sleep disordered breathing on AD biomarkers. In another recent study, cognitively normal elderly persons (aged 55–90 years) were prospectively observed for 2 years (Sharma et al., 2017). After adjusting for age, sex and BMI, the association between severity of OSA, as indicated by apnea/hypopnea index, an annual rate change of CSF Aß_42_ concentrations was found. These findings indicate that in cognitively normal older persons, OSA is associated with increased amyloid brain deposition. Repetitive arterial oxygen desaturations and/or sleep fragmentation, and known direct consequences of sleep apneas and hypopneas (Brzecka and Davies, 1993) are likely mechanisms linking OSA with MCI and AD. In a study of 38 cognitively normal persons −19 OSA patients (apnea/hypopnea index > 15/h, mean 21.2 ± 5.1/h, age 58.5 ± 4.1 years) and 19 controls of similar age—amyloid deposition in the brain was studied with Pittsburgh Compound B PET imaging (Yun et al., 2017). Higher amyloid deposition in the areas of right posterior cingulated gyrus and right temporal cortex was found in OSA patients as compared with controls, indicating the possibility of development or progression of AD as a consequence of sleep disordered breathing. Another confirmation of the link between OSA and AD was provided by a longitudinal 15-years long study of 1,667 participants (Lutsey et al., 2018). In the patients with severe OSA (with apnea/hypopnea index > 30/h)—but not in all OSA patients—there was higher risk ratio of AD dementia (1.66, 95% CI 1.06–5.18).

### Periodic limb movements during sleep and restless legs syndrome

Periodic leg movements (PLM) can also lead to sleep fragmentation. In the study cited above (Rose et al., 2011), among 37% older people with dementia, PLM index (PLMI) was ≥15/h, indicating moderate to severe form of the disease. In comparison to results of the study conducted in 455 women at the age of 82.9 years, where PLMI ≥15/h has been found in 52% of patients (Claman et al., 2006), the percentage was smaller. In the other study, including 28 patients at the age of 67.8 ± 8.7 years with AD of moderate severity (MMSE 17.8 ± 6.8 points), not receiving any treatment possibly interfering with sleeps, and examined with PSG, more frequent occurrence of PLM was not observed in comparison to healthy people at similar age (Bliwise et al., 2012). PLM is usually accompanied by restless leg syndrome (RLS). This syndrome is diagnosed on the basis of medical history. However, it has been proved that patients with AD, even in the time of mild cognitive disorders, were not able to describe the symptoms of RLS properly (Tractenberg et al., 2005). On the other hand, based on the observations of the patients, it has been stated that symptoms indicating RLS (probable diagnosis of RLS) in the course of AD occur among about 4%–5.5% of patients (Ohayon and Roth, 2002; Talarico et al., 2013). In a study of 339 patients with AD, there were 14 patients meeting the criteria of RLS (Talarico et al., 2013). The patients with concomitant RLS were younger and more apathetic than AD patients without RLS (*p* = 0.029 and *p* = 0.001, respectively). This clinical observation suggested a dysfunction of dopaminergic system in the patients with RLS in the course of AD disease. The problem of RLS in AD may be important, as there are observations from other patients' groups that up to 90% of patients with RLS have sleep disruption caused by concomitant PLM syndrome (Skalski, 2017).

### Sleep and brain glymphatic system in AD

Recent reports indicate an important relation between disrupted sleep, brain glymphatic system and AD (Mendelsohn and Larrick, 2013; Lee et al., 2015; O'Donnell et al., 2015; Krueger et al., 2016). For instance, glymphatic system consists of para-vascular channels located around blood vessels of the brain. CSF flows along para-arterial space, reaches the capillary bed and penetrates into the brain parenchyma, where it gets mixed with interstitial fluid and after collecting metabolic waste it is moved to para-venous space and then to cervical lymphatic vessels (Ratner et al., 2015). Thus, it can be stated that glymphatic system acts like the lymphatic system in the other body organs.

One of the glymphatic system functions is the removal of metabolites and neurotoxic compounds, including soluble Aβ from the CNS parenchyma (Kyrtsos and Baras, 2015; Bakker et al., 2016; Simon and Iliff, 2016). It has been demonstrated that more than half of Aβ could be removed from the brain through the glymphatic system (Iliff et al., 2012). It seems that sleep may influence glymphatic system function. During natural sleep, there is a marked increase of the brain's interstitial space as compared with wakefulness, possibly resulting from the shrinkage of astroglial cells (Mendelsohn and Larrick, 2013; Xie et al., 2013; Kress et al., 2014; O'Donnell et al., 2015). The enlargement of the extracellular space accelerates clearance processes. It has been found that in mice, the clearance of the Aβ during sleep was two-fold faster than during wakefulness (Xie et al., 2013). In the other animal study, it has been demonstrated that the speed of clearance through the glymphatic system depends also on the body posture (Lee et al., 2015). The glymphatic transport was the most efficient in the lateral position, which is the most common during sleep.

As Aβ clearance is impaired in both early and late forms of AD (Tarasoff-Conway et al., 2015), it can be assumed that there is a link between impaired glymphatic system function and AD. Experiments in animal and humans revealed diurnal oscillation of the Aβ level in the brain interstitial fluid (Musiek, 2015). Indeed, as endogenous neuronal activity influences the regional concentration of the Aβ in the interstitial fluid (Bero et al., 2011), decreased neuronal activity in some stages of sleep may cause the oscillations of the Aβ concentrations. Slow wave sleep with periodic neuronal hyperpolarization and diminished neuronal firing in some brain regions can be associated with decreased Aβ production (Musiek, 2015). Thus, altered sleep quality might contribute to the onset and progression of the AD both through impaired glymphatic clearance and disturbances in the Aβ production in case of disordered slow wave sleep.

Although the presence of glymphatic system has been proved in animal studies, there is also evidence indicating its function in humans (Kiviniemi et al., 2016). The diffusion-based MR technique called diffusion tensor image analysis along the perivascular space has been used to reflect impairment of the glymphatic system in AD patients (Taoka et al., 2017). The usefulness of diffusion tensor imaging measurements has been also shown in distinguishing patients with early-stage AD from those with subcortical ischemic vascular disease (Tu et al., 2017).

Among sleep stages, specifically slow wave sleep, exert the influence on Aβ_42_ level in the CSF. In a study encompassing 36 cognitively normal and elderly subjects, CSF Aβ_42_ levels inversely correlated with slow wave sleep duration (*r* = −0.35, *p* < 0.05), slow wave sleep % of total sleep time (*r* = −0.36, *p* < 0.05) and slow wave activity in frontal EEG leads during sleep time (*r* = −0.45, *p* < 0.01) (Varga et al., 2016). Additionally, local Aβ accumulation was found to be associated specifically with diminished slow wave activity during sleep in the low frequency range (0.6–1 Hz) (Mander et al., 2015). These findings may indicate the association between decreased clearance and/or production of Aβ and slow wave sleep deficiency.

### Final remarks

The above listed clinical and experimental observations strongly suggest bidirectional relationship between sleep and AD. Sleep disorders, such as difficulties in falling asleep, sleep disruption and altered circadian sleep-wake cycle, are typical symptoms of AD and usually escalate with progression of the disease (Bliwise et al., 1995; McCurry et al., 1999; McCurry and Ancoli-Israel, 2003; Most et al., 2012; Lim et al., 2013; Spira et al., 2013; Dos Santos et al., 2014, 2015; Spalletta et al., 2015). PSG studies reveal macro- and micro-structure sleep abnormalities in both clinical and preclinical AD (Prinz et al., 1982; Bliwise, 1993; Rauchs et al., 2008; Westerberg et al., 2012; Hita-Yañez et al., 2013; Liguori et al., 2014; Maestri et al., 2015). There is a correlation between sleep-wake rhythm disturbance and signs of cerebral Aβ deposition (Lim et al., 2013). On the other hand, sleep abnormalities may increase the risk of AD development. There are empirical data showing increased levels of Aβ_42_ in CSF after sleep deprivation (Ooms et al., 2014). There is also growing evidence showing that severe sleep disturbances caused by breathing disorders during sleep may influence AD development and progression (Dyken et al., 2004; Cooke et al., 2009; Rose et al., 2011; Daulatzai, 2013; Buratti et al., 2014; Troussière et al., 2014; Liguori et al., 2017; Sharma et al., 2017; Yun et al., 2017; Lutsey et al., 2018). In OSA patients the signs of increased amyloid deposition in the brain were observed (Sharma et al., 2017; Yun et al., 2017).

The key to understanding the link between sleep disturbances and AD development may be the function of glymphatic system. The activity of glymphatic system augments during sleep (Pistollato et al., 2016) and —to some extent —Aβ is cleared through the glymphatic system (Boespflug and Iliff, 2018). Thus, disrupted sleep may lead to glymphatic system function impairment and Aβ accumulation. Possible mechanisms of bidirectional relationship between sleep disturbances and Aβ clearance should be taken into consideration.

## Conclusion

Clinical observations indicate the likelihood of a bidirectional relationship between abnormalities of sleep and AD. Changes in sleep structure, worse sleep quality in both preclinical and symptomatic AD, correlation of cognitive impairments with sleep structure abnormalities, changes in CSF Aß concentrations induced by sleep apneas and correlating with severity of sleep disordered breathing, the influence of physiological sleep on clearance of Aβ through the glymphatic system, possible influence of impaired glymphatic system on Aβ level, and observations with the use of the newest technical equipment reflecting impairment of the glymphatic system in AD patients allow to conclude that disordered sleep may contribute to the development of AD pathology.

### Future direction of the research

1. Electrophysiologic sleep studies aimed to find early signs of risk of development of AD in target populations.
2. Development of specific procedures leading to improvement of sleep structure and quality.
3. The studies on glymphatic system in sleep breathing disorders in relation to the risk of Aβ_42_ accumulation.
4. Testing possible relationship between worsening of cognitive abilities and inflammatory state, oxidative stress, hypoxemia or vascular pathology and other neuropathological signs in AD, and TAU protein production.
5. Based on the analysis of sleep and its disorders, identify new directions of AD therapy, especially at its pre-symptomatic stages.

## Author contributions

All authors listed, have made substantial, direct and intellectual contribution to the work, and approved it for publication.

### Conflict of interest statement

GA was employed by GALLY International Biomedical Research Consulting LLC, San Antonio, Texas, USA. The other authors declare that the research was conducted in the absence of any commercial or financial relationships that could be construed as a potential conflict of interest.

## Abbreviations

AD: Alzheimer's disease
CSF: Cerebrospinal fluid
EEG: Electroencephalogram
PSG: Polysomnography
MCI: Mild cognitive impairment
PLM: Periodic leg movements
REM: Rapid eye movement
RLS: Restless leg syndrome
TST: Total sleep time.
